# Prenatal Phthalate Exposures and Neurobehavioral Development Scores in Boys and Girls at 6–10 Years of Age

**DOI:** 10.1289/ehp.1307063

**Published:** 2014-02-21

**Authors:** Roni W. Kobrosly, Sarah Evans, Amir Miodovnik, Emily S. Barrett, Sally W. Thurston, Antonia M. Calafat, Shanna H. Swan

**Affiliations:** 1 Department of Preventive Medicine, Icahn School of Medicine at Mount Sinai, New York, New York, USA; 2 Department of Obstetrics and Gynecology, and; 3 Department of Biostatistics and Computational Biology, University of Rochester School of Medicine and Dentistry, Rochester, New York, USA; 4 Division of Laboratory Sciences, National Center for Environmental Health, Centers for Disease Control and Prevention, Atlanta, Georgia, USA

## Abstract

Background: There is concern over potential neurobehavioral effects of prenatal phthalate exposures, but available data are inconsistent.

Objectives: We examined associations between prenatal urinary concentrations of phthalate metabolites and neurobehavioral scores among children.

Methods: We measured phthalate metabolite concentrations in urine samples from 153 pregnant participants in the Study for Future Families, a multicenter cohort study. Mothers completed the Child Behavior Checklist when the children were 6–10 years of age. We estimated overall and sex-specific associations between phthalate concentrations and behavior using adjusted multiple regression interaction models.

Results: In boys, concentrations of monoisobutyl phthalate were associated with higher scores for inattention (β = 0.27; 95% CI: 0.04, 0.50), rule-breaking behavior (β = 0.20; 95% CI: 0.01, 0.38), aggression (β = 0.34; 95% CI: 0.09, 0.59), and conduct problems (β = 0.39; 95% CI: 0.20, 0.58), whereas the molar sum of di(2-ethylhexyl) phthalate metabolites was associated with higher scores for somatic problems (β = 0.15; 95% CI: 0.03, 0.28). Higher monobenzyl phthalate concentrations were associated with higher scores for oppositional behavior (β = 0.16; 95% CI: 0.01, 0.32) and conduct problems (β = 0.21; 95% CI: 0.06, 0.37) in boys, but with reduced anxiety scores in girls (β = –0.20; 95% CI: –0.39, –0.01). In general, the associations reported above were close to the null among girls. Model coefficients represent the difference in the square root–transformed outcome score associated with a 1-unit increase in log-transformed metabolites.

Conclusions: Our results suggest associations between exposure to certain phthalates in late pregnancy and behavioral problems in boys. Given the few studies on this topic and methodological and population differences among studies, additional research is warranted.

Citation: Kobrosly RW, Evans S, Miodovnik A, Barrett ES, Thurston SW, Calafat AM, Swan SH. 2014. Prenatal phthalate exposures and neurobehavioral development scores in boys and girls at 6–10 years of age. Environ Health Perspect 122:521–528; http://dx.doi.org/10.1289/ehp.1307063

## Introduction

Phthalates are human-made chemicals used globally in production of commercial and industrial goods (Meeker et al. 2009). Phthalates are primarily used as plasticizers in the manufacture of flexible vinyl and polyvinyl chloride plastic, but are also used in personal care products, pharmaceuticals, textiles, medical supplies, and many other products [U.S. Environmental Protection Agency (EPA) 2007]. Because they are not covalently bound to the product matrix, phthalates can leach into the surrounding environment (U.S. EPA 2007). Exposure to phthalates can occur through ingestion, inhalation, intravenous, or dermal exposure (Meeker et al. 2009; Sathyanarayana 2008). Because of their widespread use, phthalates are ubiquitous in the environment, and nationally representative studies have demonstrated widespread exposure to multiple phthalates in the U.S. population [Centers for Disease Control and Prevention (CDC) 2012a; Silva et al. 2004a].

Concern has been raised over potential endocrine-disrupting properties of phthalates (Crisp et al. 1998; U.S. EPA 2007), particularly evidence suggesting antiandrogenic effects during prenatal development (Meeker et al. 2009; Swan et al. 2005). A less studied area of public health importance is the potential neurobehavioral effects of prenatal exposure to phthalates. Fetal brain development is tightly regulated by the maternal endocrine system (Moore and Persaud 2003). Phthalates and other endocrine-disrupting chemicals may perturb this hormonal balance and disrupt fetal brain development (Zoeller and Crofton 2000).

Two studies of prenatal phthalate exposure and neonatal behavior have focused on infants 5 days and 5 weeks after delivery, respectively (Engel et al. 2009; Yolton et al. 2011). Both studies suggest that phthalate metabolite concentrations may be associated with alertness, motor control, arousal, and other behaviors, and lend support to examining these relationships in older children. To date, two studies have examined relationships between prenatal phthalate exposure and neurobehavioral development during childhood. A 2010 study reported that among 171 children 4–9 years of age, higher prenatal urine concentrations of low-molecular weight phthalates [including di-*n*-butyl phthalate (DnBP) and diisobutyl phthalate (DiBP) (referred therein as dibutyl phthalates; DBPs) and diethyl phthalate (DEP)] were associated with higher scores for aggression, conduct problems, and depressive symptoms, but lower scores for emotional control, attention, and executive function based on parental surveys (Engel et al. 2010). Sex-stratified analyses revealed elevated coefficients among males for several of these outcome scores. More recently, Whyatt set al. (2012) reported that prenatal urinary concentrations of phthalate metabolites were associated with higher scores for emotional reactivity, somatic complaints, withdrawn behavior, and a summary measure of internalizing behavior in 3-year old children (Whyatt et al. 2012). These associations varied between boys and girls; specifically, metabolites of DBPs were associated with higher scores for emotional reactivity, somatic complaints, and withdrawn behavior in boys, whereas greater concentrations of a benzylbutyl phthalate (BzBP) metabolite were associated with higher scores for anxiety/depressive behavior, somatic complaints, and withdrawn behavior in girls. Somatic complaints and withdrawn behavior were positively associated with mono-*n*-butyl phthalate (MBP) in boys and girls.

In summary, results from the few published studies suggest associations between prenatal phthalate exposure and children’s neurodevelopment. However, given the variety of study populations, age groups, and neurobehavioral instruments employed in these studies, additional investigation is warranted. Therefore, in this analysis we examined associations between urinary prenatal phthalate metabolites concentrations and neurobehavioral scores among children 6–10 years of age.

## Methods

*Study population*. The mothers included in this analysis were originally recruited in 1999–2005 into the Study for Future Families (SFF), a multicenter pregnancy cohort study (Swan et al. 2003, 2005). A total of 961 pregnant mothers and partners were recruited from California, Minnesota, Missouri, and Iowa. Of these, 441 mothers agreed to be recontacted and were eligible for follow-up. Prenatal phthalate metabolite concentrations measured in maternal spot urine samples (obtained at mean gestational age of 26.6 ± 7.2 weeks; range, 10–39) were available for 380 of these mothers. In 2010, we recontacted SFF mothers of children born in 2000–2005. Mothers were asked to complete neurodevelopmental assessments, including the Child Behavior Checklist (CBCL); 176 mothers completed these assessments. Children were excluded from the present analysis because of incomplete covariate data (*n* = 8) or serious disability (*n* = 1). In addition, we excluded 14 children who were 5 years old at the time of assessment because the CBCL version we used is normed for children 6–10 years.

Ultimately, we analyzed data from 153 children with complete information on their mothers’ prenatal phthalate metabolite urinary concentrations, neurobehavioral scores, and covariates. Institutional review boards at all participating institutions approved all study procedures, and all participants signed informed consents for each study. The involvement of the CDC laboratory was determined not to constitute engagement in human subject research.

*Maternal urinary phthalate metabolite concentrations*. Urinary phthalate metabolites were quantified at the Division of Laboratory Sciences, National Center for Environmental Health, CDC, by solid phase extraction–high-performance liquid chromatography–isotope dilution tandem mass spectrometry. Further details of the analyses are available elsewhere (Silva et al. 2004b; Swan et al. 2005). Creatinine was measured also at CDC using an enzymatic reaction.

We included seven phthalate metabolites that are often studied for their health effects. We considered three metabolites of di(2-ethylhexyl) phthalate (DEHP): mono(2-ethylhexyl) phthalate (MEHP), mono(2-ethyl-5-hydroxyhexyl) phthalate (MEHHP), and mono(2-ethyl-5-oxohexyl) phthalate (MEOHP); and two metabolites of DBPs: monoisobutyl phthalate (MiBP) and MBP. We also considered monobenzyl phthalate (MBzP), a metabolite of BzBP, and monoethyl phthalate (MEP), a metabolite of DEP. We calculated molar sums of the three DEHP metabolites to obtain a summary measure of DEHP using a previously reported technique (Wolff et al. 2008). For example, the molar sum of the three measured DEHP metabolites was calculated by dividing the concentration of each metabolite by its molar mass and summing the results: ΣDEHP = MEHP × (1/278.34) + MEHHP × (1/294.34) + MEOHP × (1/292.33). The limit of detection (LOD) for all metabolites was between 0.95 and 1.07 ng/mL. Concentrations that were flagged as below the LOD were assigned the value of LOD divided by the square root of 2, as previously recommended (Hornung and Reed 1990).

*Neurobehavioral assessment*. Mothers rated their child’s behavior with the School-age CBCL, an extensively validated neurodevelopmental survey instrument (Achenbach and Rescorla 2001; Greenbaum et al. 2003; Kamphaus and Frick 2010). The CBCL yields seven syndrome scale scores including: Anxious/Depressed, Withdrawn/Depressed, Somatic Complaints, Social Problems, Thought Problems, Attention Problems, Rule-breaking Behavior, and Aggressive Behavior. These individual syndrome scores were combined to produce three summary measures of internalizing behavior, externalizing behavior, and a total problems score. In addition, the CBCL includes items consistent with DSM-IV (*Diagnostic and Statistical Manual of Mental Disorders, Fourth Edition*; American Psychiatric Association 2000) categories. DSM-oriented scale scores include: Affective Problems, Anxiety Problems, Somatic Problems, Attention Deficit/Hyperactivity Problems (ADHD), Oppositional/Defiant Problems, and Conduct Problems. The CBCL syndrome scales were derived empirically via factor analytic whereas the DSM-oriented scales were constructed to be consistent with DSM-IV diagnostic criteria, through expert consensus (Achenbach et al. 2003). In psychometric analysis, these DSM-oriented scales have been found to be highly reliable and displayed convergent and discriminative validity (Nakamura et al. 2009). In cases where syndrome and DSM-oriented scales overlap (e.g., the Somatic Complaints syndrome scale and the Somatic Problems DSM-oriented scale), correlations between scale types were high despite the difference in construction methodologies (Achenbach et al. 2003).

The CBCL provides raw scores for each of the scales, computed by summing parent ratings (0 = “not true,” 1 = “somewhat true,” 2 = “often true”) for the individual items of each scale. Higher raw scores indicate adverse behavior. The response categories are equally weighted, such that a child could reach a score of 8 by having four items endorsed as “often true” or eight items endorsed as “somewhat true.” The CBCL software uses individual scale and summary measure raw scores to compute *T*-scores based on a normative sample of children, accounting for age and sex. *T*-scores are calculated such that raw scores at or below the median value are assigned a value of 50 (except for the three summary measures, which were not truncated). Thus, individual scale *T*-scores are left-truncated and positively skewed.

Whether researchers should analyze CBCL raw scores or *T*-scores is a point of discussion in child development literature (Drotar et al. 1995). In this analysis, we reported the results of models using raw scores for two reasons. First, it allowed us to detect subtle associations of phthalate exposure with child behavior. Second, the use of raw scores satisfied regression model assumptions, whereas the use of *T*-scores, even with transformations, strongly violated these assumptions.

*Statistical analysis*. We calculated univariate, descriptive statistics (mean, SD, minimum, median, maximum, or frequencies if applicable) for all covariates and CBCL scores. Because of the approximate log-normal distribution of urinary phthalate metabolite concentrations, we report geometric means (with accompanying 95% CIs) as well as the 25th and 75th percentile values. We also calculated correlations between metabolites (adjusting for creatinine).

We fit linear regression models both with and without sex × phthalate interactions to examine sexual dimorphism. We reported both sets of results regardless of the significance of the interaction, as others have done (Sagiv et al. 2012). The first model estimated the overall associations between phthalate metabolite concentrations and CBCL scores, assuming a common slope for boys and girls. The second model allowed separate slopes for boys and girls by the inclusion of a sex × phthalate interaction and allowed us to estimate the significance of the interaction. Using a re-parameterization of the same model, we estimated sex-specific slopes. This model was advantageous because it permitted sex-specific estimates without the need to stratify our sample and reduce power.

In each of these models, phthalate variables were natural-log transformed. Regression assumptions (e.g., normality of residuals, homoscedasticity) were checked for all models. Given these diagnostics, we square-root transformed all CBCL raw scores to stabilize the residual variance. We adjusted for covariates thought to confound the association of interest or to strongly predict the CBCL score. Covariates included child sex, child age at time of CBCL assessment (in months), mother’s education level (at least a college education vs. less than college education), and urinary creatinine. In addition, we included a summary measure of family stress. The life events questions were derived from two validated questionnaires (Dohrenwend et al. 1978; Holmes and Rahe 1967). Both parents were asked whether any of the following life events occurred during the pregnancy: *a*) job loss, *b*) serious illness/injury in family, *c*) death of close family member, *d*) relationship difficulties with partner, *e*) legal/financial problems, or *f*) any other major event. Mothers and partners received one point for each life event that was endorsed. Life event data was available for the partners of all mothers in the study. All 12 life event items (six from each parent) were then summed to create a summary measure of prenatal stress. We did not adjust our primary models for study center, but did perform sensitivity analyses to evaluate the influence of adjustment for study center on model estimates. We did not adjust for race/ethnicity because > 90% of the final sample was non-Hispanic white. However, we repeated models after restricting the analysis to non-Hispanic white participants as a sensitivity analysis. In addition, we conducted a sensitivity analysis to examine the potential for confounding due to co-exposure to multiple phthalates by repeating regression analysis while adjusting for both molar sum DEHP and molar sum DBPs.

To ease interpretability, we calculated predicted percent change in CBCL raw scores (back-transformed into original raw score scale) between prenatal phthalate exposure at the 75th and 25th percentiles, holding covariates at their mean values. Our analysis was conducted with R version 2.15.2 (R Foundation for Statistical Computing, Vienna, Austria). For all analyses, results were considered significant at *p* ≤ 0.05.

## Results

Descriptive statistics on demographics and covariates are shown in Table 1. The final sample of 153 children included 77 boys and 76 girls (Table 1). The mean age of mothers at the time of the entry into SFF was 31.1 years (range, 18–42.3); and at the time of behavioral assessment, the mean age of the children was 102 months (8.5 years) (range, 72–126 months). Most mothers were at least college educated (85%). The mean family stress score was 1.4 (range, 0–8). Most mothers classified themselves as non-Hispanic white (92%).

**Table 1:** Descriptive statistics [mean ± SD or *n* (%)] for demographics and covariates in the final sample (*n* = 153).

Variable	Both sexes (*n *= 153)	Males (*n *= 77)	Females (*n *= 76)
Study center
California	9 (5.9)	5 (6.5)	4 (5.3)
Minnesota	57 (37.3)	31 (40.3)	26 (34.2)
Missouri	48 (31.4)	22 (28.6)	26 (34.2)
Iowa	39 (25.5)	19 (24.7)	20 (26.3)
Mother’s race/ethnicity
Hispanic/Latina	4 (2.6)	1 (1.3)	3 (3.9)
Non-Hispanic white	141 (92.2)	72 (93.5)	69 (90.8)
Black	5 (3.3)	3 (3.9)	2 (2.6)
Asian	3 (2.0)	1 (1.3)	2 (2.6)
Child age (months)	102.0 ± 12.0	101.8 ± 12.1	102.3 ± 12.0
Mother’s education
< College	23 (15.0)	9 (11.7)	14 (18.4)
≥ College	130 (85.0)	68 (88.3)	62 (81.6)
Mother’s age at enrollment (years)	31.1 ± 4.9	31.1 ± 4.8	31.1 ± 5.1
Family stress during pregnancy	1.4 ± 1.7	1.4 ± 1.7	1.4 ± 1.7

The phthalate metabolite concentrations we observed (Table 2) were comparable to those of a nationally representative sample of women of reproductive age (Kobrosly et al. 2012). As expected, all phthalate metabolite concentrations were positively skewed. Levels appeared to be similar between boys and girls except for MEP concentrations, which were slightly higher among boys. The proportions of metabolite observations below the LOD were 5% for all metabolites other than MEHP (21% < LOD) and MiBP (18% < LOD). Correlations between DEHP metabolites were high (Pearson *r* = 0.72–0.98), and the correlation between the DBP metabolites (MiBP and MBP) was moderate (Pearson *r* = 0.51) (see Supplemental Material, Table S1).

**Table 2:** Descriptive statistics for CBCL scores and phthalate metabolites in the final sample *n* = 153).

Variable	Both sexes		Males		Females	
	Mean ± SD or GM (95% CI)	Median (min–max) or 25th, 75th percentiles	Mean ± SD or GM (95% CI)	Median (min–max) or 25th, 75th percentiles	Mean ± SD or GM (95% CI)	Median (min–max) or 25th, 75th percentiles
CBCL syndrome scales (raw scores)
Anxious/Depressed	3.1 ± 2.9	2 (0–12)	3.2 ± 2.9	2 (0–11)	2.9 ± 3.0	2 (0–12)
Withdrawn/Depressed	1.0 ± 1.7	0 (0–10)	1.2 ± 1.8	0 (0–8)	0.9 ± 1.5	0 (0–10)
Somatic Complaints	1.4 ± 1.7	1 (0–8)	1.4 ± 1.7	1 (0–8)	1.3 ± 1.7	1 (0–8)
Social Problems	2.0 ± 2.1	1 (0–12)	2.3 ± 2.4	1 (0–12)	1.8 ± 1.7	1 (0–7)
Thought Problems	1.8 ± 2.0	1 (0–11)	2.2 ± 2.3	2 (0–11)	1.4 ± 1.5	1 (0–7)
Attention Problems	3.7 ± 3.4	3 (0–18)	4.7 ± 3.6	4 (0–18)	2.8 ± 2.9	2 (0–12)
Rule-breaking Behavior	1.3 ± 1.6	1 (0–9)	1.7 ± 1.9	1 (0–9)	0.9 ± 1.1	0.5 (0–5)
Aggressive Behavior	3.6 ± 3.7	3 (0–16)	4.3 ± 4.2	3 (0–16)	2.9 ± 3.0	2 (0–14)
Internalizing behavior	5.4 ± 4.9	4 (0–23)	5.7 ± 5.1	5 (0–23)	5.1 ± 4.7	4 (0–21)
Externalizing behavior	4.9 ± 4.9	3 (0–23)	6.0 ± 5.7	4 (0–23)	3.8 ± 3.6	3 (0–16)
Total problems	21.0 ± 15.0	17 (1–70)	23.8 ± 17.2	19 (2–70)	18.0 ± 11.9	15 (1–49)
CBCL DSM scales (raw scores)
Affective Problems	1.2 ± 1.7	1 (0–8)	1.6 ± 2.0	1 (0–8)	0.9 ± 1.3	0 (0–6)
Anxiety Problems	1.5 ± 1.8	1 (0–8)	1.7 ± 1.8	1 (0–7)	1.4 ± 1.8	1 (0–8)
Somatic Problems	0.8 ± 1.5	0 (0–7)	0.8 ± 1.5	0 (0–7)	0.8 ± 1.5	0 (0–7)
ADHD Problems	3.1 ± 2.7	3 (0–11)	3.9 ± 2.8	3 (0–11)	2.3 ± 2.3	2 (0–10)
Oppositional/Defiant Problems	1.9 ± 1.9	1 (0–8)	2.3 ± 2.1	2 (0–8)	1.5 ± 1.6	1 (0–6)
Conduct Problems	1.3 ± 2.0	0 (0–12)	1.9 ± 2.5	1 (0–12)	0.6 ± 1.0	0 (0–3)
Phthalate metabolites^*a,b*^
MEHP	3.65 (2.89, 4.61)	1.1, 9.9	3.52 (2.49, 4.98)	0.8, 6.7	3.79 (2.79, 5.15)	1.2, 10.7
MEHHP	13.04 (10.28, 16.55)	6.1, 24.2	12.49 (9.0, 17.33)	5.5, 24.1	13.63 (9.67, 19.21)	6.3, 27.1
MEOHP	11.50 (9.16, 14.44)	5.1, 22.0	11.19 (8.15, 15.38)	4.7, 21.5	11.82 (8.56, 16.32)	5.2, 24.7
MiBP	2.34 (1.98, 2.78)	1.0, 4.8	2.34 (1.83, 2.99)	1.1, 5.1	2.35 (1.86, 2.97)	1.0, 4.7
MBP	13.61 (11.52, 16.07)	7.8, 29.4	13.36 (10.54, 16.94)	7.3, 29.6	13.86 (11.0, 17.46)	8.4, 28.1
MBzP	6.59 (5.34, 8.15)	3.4, 16.3	6.43 (4.80, 8.61)	2.7, 15.5	6.76 (4.99, 9.16)	4.0, 18.4
MEP	81.01 (62.09, 105.70)	26.2, 231.0	85.66 (59.27, 123.82)	31.0, 307.5	76.56 (52.35, 111.96)	23.2, 168.3
Abbreviations: max, maximum; min, minimum. ^***a***^Limit of detection for all metabolites was between 0.95 and 1.07 ng/mL. ^***b***^Number of metabolite observations below the LOD in final sample of both sexes: MEHP: *n *= 32 (20.9%), MEHHP: *n *= 4 (2.6%), MEOHP: *n *= 5 (3.3%), MiBP: *n *= 27 (17.6%), MBP: *n *= 4 (2.6%), MBzP: *n *= 6 (3.9%), MEP: *n*= 1 (0.7%).						

Using chi-square tests and *t*-tests, we compared covariate and phthalate concentrations between our final sample (*n* = 153) and data from 359 of the 441 mothers who were eligible for follow-up after the first phase of SFF (see Supplemental Material, Table S2). These groups did not differ significantly with regard to baseline family stress (*p* = 0.15) or any phthalate metabolite concentrations. However, the children in the final sample had mothers with higher education (*p* = 0.005) and older age (*p* = 0.04), and were more likely to be non-Hispanic white (*p* = 0.03).

Raw CBCL scores for each category were positively skewed (Table 2). Male children exhibited higher raw scores (adverse behavior) than females on all categories of the syndrome scale. The same was true for most categories of the DSM-oriented scale. In addition to the raw scores, we calculated *T*-scores for the CBCL syndrome and DSM-oriented scales that indicate how scores for the study population compare with normative scores for children of the same age and sex. Mean percentiles across all outcomes ranged from 59.79% to 65.5%, which suggests that our study population had a higher prevalence of behavioral problems compared with the CBCL normative sample.

*DEHP molar sum*. Table 3 presents associations between prenatal phthalate metabolite concentrations (for molar sum DEHP and other metabolites) and CBCL syndrome scale scores in a combined sample of boys and girls, as well as sex-stratified samples. Table 4 provides this information for the CBCL DSM-oriented scale scores. Somatic Complaints scores were positively associated with urinary concentrations of summed DEHP metabolites (β = 0.10; 95% CI: 0.01, 0.20, for the association between a 1-unit increased in the ln-transformed metabolite concentration and the square root of the outcome score) in the combined sample of boys and girls (Table 3). Sex-specific analyses revealed that boys were driving these associations (β = 0.15; 95% CI: 0.03, 0.28); the result corresponds to a 153% increase in the raw score between the 25th and 75th percentile of DEHP metabolites (holding all covariates constant at their mean value) This association among girls was null, and the interaction *p*-value for the difference between boys and girls was not significant (*p*_int_ = 0.25). Summed DEHP metabolites were associated with lower Anxious/Depressed syndrome scores in girls (β = –0.21; 95% CI: –0.38, –0.04; corresponding to a 37% reduction from the 25th to the 75th percentile of exposure) but there was no association in boys (*p*_int_ = 0.04). Similarly, we observed negative associations of summed DEHP metabolites with the Anxiety Problems DSM-oriented score in girls (β = –0.21; 95% CI: –0.35, –0.06; corresponding to 51% reduction from the 25th to 75th percentile) but not boys (*p*_int_ = 0.01) (Table 4).

**Table 3:** Regression phthalate metabolite coefficients (with 95% CIs) of models predicting CBCL syndrome scale scores, according to child sex.^*a*^

CBCL syndrome scales	Sex	ln(∑DEHP)	*p*_int_^*b*^	ln(MiBP)	*p*_int_	ln(MBP)	*p*_int_	ln(MBzP)	*p*_int_	ln(MEP)	*p*_int_
Anxious/Depressed	Overall	–0.09 (–0.22, 0.03)		0.04 (–0.16, 0.24)		–0.06 (–0.27, 0.15)		–0.13 (–0.28, 0.03)		–0.05 (–0.16, 0.06)
	Male	0.02 (–0.15, 0.18)		0.11 (–0.13, 0.34)		0.01 (–0.25, 0.26)		–0.06 (–0.25, 0.13)		–0.04 (–0.18, 0.10)
	Female	–0.21 (–0.38, –0.04)*	0.04	–0.03 (–0.29, 0.22)	0.35	–0.14 (–0.40, 0.12)	0.35	–0.20 (–0.39, –0.01)*	0.24	–0.06 (–0.20, 0.08)	0.81
Withdrawn/Depressed	Overall	0.01 (–0.10, 0.11)		–0.03 (–0.19, 0.14)		–0.02 (–0.19, 0.15)		–0.06 (–0.18, 0.07)		–0.08 (–0.16, 0.01)
	Male	0.04 (–0.09, 0.18)		–0.01 (–0.21, 0.18)		0.02 (–0.19, 0.23)		0.02 (–0.14, 0.17)		–0.05 (–0.17, 0.06)
	Female	–0.03 (–0.18, 0.11)	0.39	–0.04 (–0.25, 0.17)	0.82	–0.06 (–0.27, 0.15)	0.54	–0.13 (–0.29, 0.02)	0.12	–0.10 (–0.22, 0.02)	0.56
Somatic Complaints	Overall	0.07 (–0.04, 0.17)		–0.05 (–0.21, 0.11)		–0.10 (–0.27, 0.07)		–0.04 (–0.16, 0.08)		–0.01 (–0.10, 0.07)
	Male	0.1 (–0.04, 0.23)		–0.03 (–0.23, 0.16)		–0.07 (–0.28, 0.13)		0.004 (–0.15, 0.16)		0.03 (–0.08, 0.15)
	Female	0.03 (–0.10, 0.17)	0.48	–0.07 (–0.28, 0.13)	0.74	–0.13 (–0.34, 0.08)	0.66	–0.08 (–0.24, 0.07)	0.38	–0.06 (–0.18, 0.05)	0.21
Social Problems	Overall	–0.07 (–0.18, 0.04)		0.07 (–0.10, 0.24)		–0.04 (–0.22, 0.14)		–0.04 (–0.17, 0.09)		–0.08 (–0.17, 0.01)
	Male	–0.04 (–0.18, 0.10)		0.18 (–0.02, 0.37)		0.02 (–0.19, 0.24)		0.06 (–0.10, 0.22)		–0.06 (–0.18, 0.05)
	Female	–0.11 (–0.26, 0.03)	0.44	–0.06 (–0.27, 0.16)	0.06	–0.10 (–0.32, 0.11)	0.33	–0.14 (–0.30, 0.02)	0.05	–0.09 (–0.21, 0.03)	0.71
Thought Problems	Overall	0.03 (–0.08, 0.14)		0.12 (–0.05, 0.28)		–0.02 (–0.20, 0.16)		–0.05 (–0.18, 0.08)		–0.07 (–0.16, 0.02)
	Male	0.07 (–0.07, 0.22)		0.15 (–0.05, 0.35)		–0.01 (–0.23, 0.20)		–0.06 (–0.22, 0.11)		–0.10 (–0.21, 0.02)
	Female	–0.02 (–0.17, 0.12)	0.31	0.07 (–0.15, 0.29)	0.51	–0.03 (–0.25, 0.19)	0.89	–0.04 (–0.20, 0.12)	0.87	–0.04 (–0.16, 0.08)	0.47
Attention Problems	Overall	0.08 (–0.04, 0.21)		0.20 (0.01, 0.39)*		0.06 (–0.14, 0.26)		–0.05 (–0.20, 0.10)		–0.04 (–0.14, 0.07)
	Male	0.07 (–0.10, 0.23)		0.27 (0.04, 0.50)*		0.12 (–0.12, 0.37)		0.003 (–0.18, 0.19)		–0.08 (–0.22, 0.06)
	Female	0.10 (–0.07, 0.26)	0.77	0.12 (–0.12, 0.36)	0.29	–0.01 (–0.26, 0.25)	0.38	–0.10 (–0.29, 0.08)	0.35	0.01 (–0.13, 0.14)	0.35
Rule-breaking Behavior	Overall	0.002 (–0.10, 0.10)		0.09 (–0.06, 0.25)		0.08 (–0.08, 0.25)		–0.01 (–0.13, 0.11)		0.01 (–0.08, 0.09)
	Male	0.04 (–0.09, 0.17)		0.20 (0.01, 0.38)*		0.14 (–0.05, 0.34)		0.08 (–0.07, 0.23)		0.02 (–0.09, 0.13)
	Female	–0.04 (–0.18, 0.09)	0.37	–0.04 (–0.23, 0.16)	0.04	0.02 (–0.19, 0.22)	0.28	–0.10 (–0.25, 0.05)	0.06	–0.01 (–0.12, 0.10)	0.67
Aggressive Behavior	Overall	–0.05 (–0.19, 0.09)		0.24 (0.03, 0.45)*		0.03 (–0.19, 0.25)		0.10 (–0.07, 0.26)		–0.06 (–0.18, 0.05)
	Male	–0.02 (–0.20, 0.15)		0.34 (0.09, 0.59)*		0.12 (–0.15, 0.39)		0.19 (–0.01, 0.40)		–0.04 (–0.19, 0.11)
	Female	–0.08 (–0.26, 0.10)	0.64	0.12 (–0.14, 0.39)	0.16	–0.07 (–0.34, 0.21)	0.24	–0.002 (–0.21, 0.20)	0.11	–0.08 (–0.23, 0.07)	0.70
Internalizing behavior	Overall	–0.02 (–0.17, 0.13)		0.02 (–0.21, 0.25)		–0.08 (–0.33, 0.16)		–0.13 (–0.30, 0.05)		–0.05 (–0.18, 0.07)
	Male	0.09 (–0.1, 0.28)		0.09 (–0.18, 0.37)		–0.01 (–0.30, 0.29)		–0.04 (–0.25, 0.18)		–0.02 (–0.19, 0.14)
	Female	–0.14 (–0.34, 0.06)	0.08	–0.07 (–0.37, 0.22)	0.32	–0.16 (–0.46, 0.14)	0.37	–0.22 (–0.44, 0)*	0.17	–0.08 (–0.25, 0.08)	0.60
Externalizing behavior	Overall	–0.04 (–0.18, 0.11)		0.20 (–0.02, 0.42)		0.08 (–0.16, 0.31)		0.07 (–0.10, 0.24)		–0.03 (–0.15, 0.09)
	Male	–0.003 (–0.19, 0.19)		0.32 (0.06, 0.58)*		0.17 (–0.12, 0.45)		0.18 (–0.03, 0.40)		–0.02 (–0.18, 0.14)
	Female	–0.08 (–0.27, 0.12)	0.55	0.06 (–0.22, 0.34)	0.10	–0.02 (–0.31, 0.27)	0.26	–0.04 (–0.25, 0.17)	0.09	–0.05 (–0.21, 0.11)	0.79
Total problems	Overall	–0.01 (–0.22, 0.20)		0.27 (–0.05, 0.58)		–0.004 (–0.34, 0.33)		–0.05 (–0.30, 0.19)		–0.12 (–0.29, 0.05)
	Male	0.07 (–0.19, 0.34)		0.42 (0.05, 0.80)*		0.12 (–0.29, 0.53)		0.10 (–0.20, 0.40)		–0.11 (–0.34, 0.11)
	Female	–0.10 (–0.37, 0.18)	0.33	0.07 (–0.33, 0.47)	0.13	–0.14 (–0.55, 0.28)	0.29	–0.21 (–0.51, 0.10)	0.10	–0.12 (–0.35, 0.10)	0.95
^***a***^Outcomes are square root–transformed raw CBCL syndrome scores. Overall estimates are from models adjusted for child sex, child age (months), mother’s education (at least college education, yes or no), creatinine, and family stress score. Sex-specific estimates and interaction *p*-values are from models that include interactions between sex and ln-metabolite concentrations, with adjustment for same covariates. ^***b***^*p*_int_, interaction *p*-value. *p ≤ 0.05.											

**Table 4:** Regression phthalate metabolite coefficients (with 95% CIs) of models predicting CBCL DSM-oriented scale scores, according to child sex.^*a*^

CBCL DSM-oriented scales	Sex	ln(∑DEHP)	*p*_int_^*b*^	ln(MiBP)	*p*_int_	ln(MBP)	*p*_int_	ln(MBzP)	*p*_int_	ln(MEP)	*p*_int_
Affective Problems	Overall	–0.06 (–0.17, 0.04)		0.03 (–0.14, 0.19)		–0.17 (–0.34, 0)		–0.12 (–0.25, 0.01)		–0.10 (–0.19, –0.01)*
	Male	–0.04 (–0.18, 0.10)		0.07 (–0.13, 0.27)		–0.15 (–0.36, 0.07)		–0.09 (–0.24, 0.07)		–0.12 (–0.24, –0.01)*
	Female	–0.09 (–0.23, 0.06)	0.62	–0.02 (–0.24, 0.19)	0.46	–0.20 (–0.41, 0.02)	0.69	–0.15 (–0.31, 0.01)	0.49	–0.08 (–0.19, 0.04)	0.57
Anxiety Problems	Overall	–0.08 (–0.19, 0.03)		0.02 (–0.15, 0.19)		–0.03 (–0.21, 0.14)		–0.11 (–0.24, 0.02)		–0.03 (–0.12, 0.07)
	Male	0.04 (–0.10, 0.18)		0.11 (–0.09, 0.31)		0.06 (–0.16, 0.27)		–0.04 (–0.20, 0.12)		–0.01 (–0.13, 0.11)
	Female	–0.21 (–0.35, –0.06)*	0.01	–0.09 (–0.31, 0.12)	0.10	–0.13 (–0.35, 0.09)	0.15	–0.19 (–0.35, –0.03)*	0.13	–0.04 (–0.16, 0.08)	0.69
Somatic Problems	Overall	0.10 (0.01, 0.20)*		–0.03 (–0.18, 0.12)		–0.07 (–0.22, 0.09)		–0.01 (–0.13, 0.10)		0.02 (–0.06, 0.10)
	Male	0.15 (0.03, 0.28)*		–0.02 (–0.20, 0.16)		–0.01 (–0.20, 0.18)		0.04 (–0.10, 0.19)		0.08 (–0.02, 0.19)
	Female	0.06 (–0.07, 0.18)	0.25	–0.05 (–0.24, 0.15)	0.81	–0.13 (–0.32, 0.07)	0.31	–0.07 (–0.21, 0.08)	0.22	–0.04 (–0.15, 0.06)	0.07
ADHD Problems	Overall	0.03 (–0.08, 0.15)		0.10 (–0.08, 0.27)		0.02 (–0.16, 0.21)		0.02 (–0.12, 0.15)		0.02 (–0.08, 0.11)
	Male	0.01 (–0.13, 0.16)		0.13 (–0.08, 0.34)		0.07 (–0.16, 0.29)		0.06 (–0.11, 0.23)		–0.03 (–0.16, 0.09)
	Female	0.05 (–0.10, 0.21)	0.68	0.06 (–0.16, 0.28)	0.59	–0.02 (–0.25, 0.21)	0.52	–0.02 (–0.19, 0.15)	0.43	0.07 (–0.05, 0.20)	0.19
Oppositional/Defiant Problems	Overall	–0.02 (–0.13, 0.09)		0.19 (0.02, 0.35)*		0.04 (–0.13, 0.22)		0.08 (–0.04, 0.21)		–0.04 (–0.13, 0.05)	
	Male	0.003 (–0.14, 0.15)		0.27 (0.07, 0.46)*		0.14 (–0.07, 0.36)		0.16 (0.01, 0.32)*		–0.02 (–0.13, 0.10)	
	Female	–0.05 (–0.19, 0.10)	0.57	0.09 (–0.12, 0.30)	0.14	–0.06 (–0.28, 0.15)	0.10	0.0002 (–0.16, 0.16)	0.10	–0.06 (–0.18, 0.06)	0.57
Conduct Problems	Overall	0.01 (–0.10, 0.11)		0.22 (0.05, 0.38)*		0.19 (0.02, 0.37)*		0.07 (–0.06, 0.2)		–0.01 (–0.10, 0.08)
	Male	0.07 (–0.07, 0.21)		0.39 (0.20, 0.58)*		0.36 (0.15, 0.56)*		0.21 (0.06, 0.37)*		0.06 (–0.06, 0.18)
	Female	–0.07 (–0.21, 0.08)	0.14	–0.004 (–0.21, 0.20)	< 0.001	0.02 (–0.19, 0.23)	0.01	–0.07 (–0.23, 0.09)	< 0.001	–0.08 (–0.20, 0.04)	0.08
^***a***^Outcomes are square root–transformed raw CBCL syndrome scores. Overall estimates are from models adjusted for child sex, child age (months), mother’s education (at least college education, yes or no), creatinine, and family stress score. Sex-specific estimates and interaction *p*-values are from models that include interactions between sex and ln-metabolite concentrations, with adjustment for same covariates. ^***b***^*p*_int_, interaction *p*-value. *p ≤ 0.05.											

*Metabolites of DBPs*. We observed multiple associations of ln-transformed urinary concentrations of MiBP and MBP, the two DBPs metabolites, with outcome scores in the combined sample of boys and girls. Among CBCL syndrome score outcomes (Table 3), MiBP concentrations were associated with higher scores for Attention Problems (β = 0.20; 95% CI: 0.01, 0.39) and Aggressive Behavior (β = 0.24; 95% CI: 0.03, 0.45). Among DSM-oriented scale scores, MiBP concentrations were associated with higher Oppositional/Defiant Problems scores (β = 0.19; 95% CI: 0.02, 0.35), and both DBPs were associated with higher Conduct Problems scores (MiBP: β = 0.22; 95% CI: 0.05, 0.38; MBP: β = 0.19; 95% CI: 0.02, 0.37).

Associations that were statistically significant in the total sample were stronger in boys than in girls, though differences were significant for Conduct Problems scores only [e.g., for MiBP: β = 0.39; 95% CI: 0.20, 0.58 in boys (corresponding to a 260% increase from the 25th to 75th percentile) vs. β = 0.00; 95% CI: –0.21, 0.20 in girls, *p*_int_ = < 0.001]. In addition, MiBP concentrations were positively associated with scores for Rule-breaking Behavior (β = 0.20; 95% CI: 0.01, 0.38), externalizing behavior (β = 0.32; 95% CI: 0.06, 0.58), and total problems (β = 0.42; 95% CI: 0.05, 0.8) in boys, whereas corresponding estimates in girls were close to the null (*p*_int_ = 0.04, 0.10, and 0.13, respectively) (Table 3). We observed no associations of either MiBP or MBP with any CBCL score in girls.

*BzBP metabolite: MBzP*. There were no statistically significant associations between MBzP and CBCL scores in the population as a whole, but there was some evidence of sex-specific relationships. Among boys, MBzP concentrations were associated with higher Oppositional/Defiant behavior scores [β = 0.16; 95% CI: 0.01, 0.32 (a 50% increase from the 25th to 75th percentile)] and Conduct Problems [β = 0.21; 95% CI: 0.06, 0.37 (a 92% increase from the 25th to 75th percentile)] in boys, whereas associations were close to the null for girls (*p*_int_ = 0.10 and 0.01, respectively) (Table 4). In girls, MBzP concentrations were associated with lower scores for syndrome scale Anxious/Depressed [β = –0.20; 95% CI: –0.39, –0.01 (a 37% decrease from the 25th to 75th percentile)], internalizing behavior [β = –0.22; 95% CI: –0.44, 0 (a 30% decrease from the 25th to 75th percentile)], and DSM Anxiety Problems scores [β = –0.19; 95% CI: –0.35, –0.03 (a 51% decrease from the 25th to 75th percentile)], whereas associations were close to the null for boys (*p*_int_ = 0.24, 0.17, and 0.13, respectively) (Tables 3 and 4).

*DEP metabolite: MEP*. Inverse associations between MEP urinary concentrations and the DSM Affective Problems score were seen when both sexes were combined and among boys: β = –0.10 (95% CI: –0.19, –0.01) and β = –0.12 (95% CI: –0.24, –0.01), respectively. However, the association was null among girls (β = –0.08; 95% CI: –0.19, 0.04) and the interaction *p*-value for the difference between boys and girls was not significant (*p*_int_ = 0.57). None of the CBCL scores were significantly associated with MEP concentrations among girls.

*Sensitivity analyses*. Our sensitivity analysis revealed that the direction and magnitude of significant and nonsignificant associations were not materially affected by adjusting for other phthalate metabolites (data not shown). In another sensitivity analysis, we found no substantial differences in our results when we adjusted for study center; the largest change in association magnitude was for the association between MiBP and attention problems in the combined sample of boys and girls (β = 0.20; 95% CI: 0.01, 0.39 in original analysis became β = 0.23; 95% CI: 0.03, 0.43). Finally, when we restricted our main analysis to non-Hispanic white mothers, although the overall interpretation of the results did not change, a few of the beta estimates changed appreciably. Specifically, among the combined sample of boys and girls and focusing on MiBP, three CBCL scores lost significance: Attention Problems [β = 0.20 (95% CI: 0.01, 0.39) in original analysis became β = 0.19 (95% CI: –0.01, 0.40)], Aggressive Behavior [β = 0.24 (95% CI: 0.03, 0.45) in original analysis became β = 0.21 (95% CI: –0.01, 0.43)], and the DSM Oppositional/Defiant Problems score [(β = 0.19; 95% CI: 0.02, 0.35) in original analysis became β = 0.17 (95% CI: 0, 0.34)]. The association between MBP and DSM Affective Problems score strengthened and attained statistical significance [β = –0.17 (95% CI: –0.34, 0) in original analysis became β = –0.21 (95% CI: –0.39, –0.03)]. Among MBzP coefficients, three associations with the following categories became more negative and gained statistical significance: syndrome scale Anxious/Depressed [β = –0.13 (95% CI: –0.28, 0.03) in original analysis became β = –0.17 (95% CI: –0.32, –0.01)], DSM-oriented Affective Problems [β = –0.12 (95% CI: –0.25, 0.01) in original analysis became β = –0.14 (95% CI: –0.27, –0.01)], and DSM-oriented Anxiety Problems [β = –0.11 (95% CI: –0.24, 0.02) in original analysis became β = –0.14 (95% CI: –0.27, 0)]. Finally, the association between MEP and the DSM Affective Problems score became more negative and gained significance [β = –0.10 (95% CI: –0.19, –0.01) in original analysis became β = –0.09 (95% CI: –0.19, 0)].

## Discussion

In this analysis we examined associations between maternal phthalate metabolite urinary concentrations during pregnancy and neurobehavioral development in their children at ages of 6–10 years. Our analyses focused on metabolites of five phthalate esters to which exposure is particularly widespread. Our results indicated a variety of associations between prenatal phthalate exposures and behavior syndrome scores based on maternal reports, and many of the associations appeared to differ between boys and girls.

A primary source of DEHP exposure is through consumption of food, with contamination likely occurring through packing, storage and processing (Clark et al. 2002; Fromme et al. 2007; Rudel et al. 2011). DBPs are found in diverse sources such as consumer plastics, personal care products, and DnBP is also used in the enteric coating of some medications (Hernandez-Diaz et al. 2009). Exposure to BzBP occurs primarily through diet, as well as through products such as adhesives, vinyl tile, sealants, car care products, and some personal care products (CDC 2012b). DEP exposure occurs primarily through use of personal care products, cosmetics, and perfumed products (Koniecki et al. 2011). Although the production of various phthalates is changing (most notably the reduction of DEHP and the increase of diisononyl phthalate in the European Union), exposure to the phthalates we considered remains widespread (Goen et al. 2011).

In our analysis, prenatal concentrations of DEHP metabolites were associated with greater somatic complaints in children. Somatic complaints include general physical symptoms such as headaches, stomach aches, feeling jittery, and muscular tension; the presence of these symptoms has been previously linked with poorer school performance in children (Hughes et al. 2008). We also noted lower symptoms of anxiety among girls whose mothers had higher concentrations of DEHP metabolites. To the best of our knowledge, ours is the first analysis to report an association between prenatal DEHP exposure and childhood neurobehavior scores assessed through a standardized instrument.

Focusing on magnitudes of association, our strongest finding was the association of prenatal concentration of DBP metabolites with a range of conduct-related behavior scores (e.g., Attention Problems, Rule-breaking Behavior, Aggressive Behavior, Oppositional/Defiant Problems) in boys. One prior analysis of 3-year-old children reported that prenatal urinary concentrations of individual metabolites of DBPs (both DnBP and DiBP) were associated with more negative internalizing behavior (a summary score encompassing Somatic Complaints, Anxious/Depressed behavior, and Withdrawn/Depressed behavior) (Whyatt et al. 2012). Our results did not support this prior finding. Although our study and Whyatt et al.’s (2012) study employed the CBCL inventory, we used the edition targeted to children of 6–10 years of age, whereas their study analyzed the edition for 1.5- to 5-year-olds. Our finding is consistent with another analysis that described associations of a measure of low-molecular-weight phthalates (which included metabolites of DBPs) with higher scores on the Behavior Assessment System for Children’s externalizing behavior category (e.g., increased aggression, inattention, and conduct problems) in 4- to 9-year-old boys but not among girls (Engel et al. 2010).

The associations we report generally differed between boys and girls, which is consistent with the two prior studies. Higher prenatal urine concentrations of MBzP were associated with higher scores for Oppositional/Defiant Problems and Conduct Problems in boys, but not girls, and with lower scores for Anxiety Problems in girls but not boys. Whyatt et al. (2012) reported that MBzP concentrations were associated with higher scores for internalizing behaviors such as Anxious/Depressed, Withdrawn/Depressed, and Somatic Complaints, in girls, but not boys. Finally, we found an association of greater MEP urinary concentrations with fewer Affective Problems symptoms (i.e., problems relating to mood, such as depression, mania, anxiety) in a combined sample of boys and girls.

To our knowledge, this is the third analysis to date of prenatal phthalate exposure and child behavioral development (Engel et al. 2010; Whyatt et al. 2012). Differences in study methods and populations may help explain differences in findings among the studies. Women in our study were older at pregnancy, mostly non-Hispanic white, and had higher educational attainment than women in the other studies. When behavioral assessment occurred, our study’s children were 6–10 years of age, compared with 4–9 years (Engel et al. 2010) and 3 years of age (Whyatt et al. 2012). There were also key differences in study design. Our study employed a multicenter design, so the population may be more geographically representative of the U.S. population. Specifically, our study sample consisted of California, Minnesota, Missouri, and Iowa, whereas the two prior studies drew subjects from New York City. SFF women gave urine samples at somewhat earlier gestational ages (mean, 26.6 weeks) than those in the studies by Engel (31.2 weeks) and Whyatt (33.1 weeks). Both our analysis and that by Whyatt et al. measured behavior with the CBCL, whereas Engel et al. relied on the Behavior Rating Inventory of Executive Function and the Behavior Assessment System for Children–Parent Rating Scales.

Mechanisms that have been proposed for potential effects of prenatal phthalates on neurodevelopment are complex and uncertain. One previously suggested mechanism involves phthalate alteration of thyroid system function (Engel et al. 2010). In humans, thyroid hormones play a crucial role in neurodevelopment (Miller et al. 2009), consistent with the finding that congenital thyroid disorders are linked with neurodevelopmental deficits in children (Oerbeck et al. 2003). Effects of phthalate intake on thyroid hormone levels have long been observed in animal models (Hinton et al. 1986). Urinary concentrations of DEHP metabolites were inversely associated with serum thyroid measures, including total and free thyroxine, total triiodothyronine, and thyroglobulin, in an analysis of data from adult and adolescent participants in the 2007–2008 U.S. NHANES (National Health and Nutrition Examination Survey) (Meeker and Ferguson 2011). This analysis also demonstrated an inverse association between mono(3-carboxypropyl) phthalate (a nonspecific metabolite of several high molecular weight phthalates and a minor metabolite of DnBP) and total and free thyroxine. In a sample study of 76 Taiwanese pregnant women, urinary concentrations of DBPs were negatively associated with thyroxin and free thyroxin (Huang et al. 2007).

A recent study of 845 Danish children 4–9 years of age in Copenhagen found associations between childhood urinary phthalate metabolite concentrations and thyroid hormone and IGF-1 (insulin-like growth factor 1) (Boas et al. 2010). Specifically, metabolites of DEHP were negatively associated with IGF-1 among boys, whereas a cumulative measure of phthalate exposure (encompassing DEHP metabolites, MEP, and MBP) was negatively associated with triiodothyronine in girls. These sexually dimorphic associations may provide a possible explanation for the observed sexually dimorphic behavioral effects. In addition to thyroid system dysfunction, it has been suggested that gestational exposure to phthalates leads to aberrant development of the midbrain dopaminergic system and hyperactivity in rats (Tanida et al. 2009). Dopaminergic signaling is known to play a role in the pathogenesis of ADHD (Del Campo et al. 2011), suggesting that phthalate-induced alteration to this pathway may account for our observed association between phthalates and attentional behavior scores.

Our observation that individual phthalate metabolites differentially correlate with behavior is consistent with several other studies (Engel et al. 2010; Swan et al. 2005; Whyatt et al. 2012). This may be explained by differences in routes of exposure or rates of metabolism and excretion (Frederiksen et al. 2007). These inconsistencies might also partly reflect random error, because we estimated a very large number of associations, and at least some may have occurred by chance.

Our analysis also has some limitations. First, we obtained only one prenatal urine sample. Had we been able to analyze several samples collected at different time points, we might have been able to estimate fetal exposure to phthalates more accurately or possibly identify a critical developmental window with regard to phthalate effects and child behavior. Several studies have shown that the intraclass correlation coefficient (ICC)—a measure of reproducibility—of phthalate metabolite urinary concentrations varies across metabolites (Adibi et al. 2008; Hauser et al. 2004; Peck et al. 2010). Collectively, these studies suggest that urinary concentration ICCs are low for DEHP metabolites and moderate for the metabolites of DBPs, with some conflicting results for MEP. This type of misclassification of phthalate exposure (error due to low reproducibility) would be expected to bias our findings toward the null. However, other potential sources of bias, such as uncontrolled confounding, could cause bias in various directions.

Second, the clinical significance of the associations we estimated is unknown. The square root–transformed raw scores of the CBCL do not correspond to clinically relevant developmental outcomes, but instead were intended to capture statistical differences in a research context. However, it is understood that although small shifts in the population distribution of continuous health measures may not be meaningful at the individual level, these can take on considerable public health significance at the tails of the distribution, particularly when shifts are a consequence of common exposures (Bellinger 2004).

Third, we were unable to adjust for several potentially confounding variables such as mother’s race. We chose to statistically adjust only for covariates with sufficient variability. Adjusting for covariates with little variability would have produced imprecise estimates and decrease the degrees of freedom (which were critical, considering our small sample size). We cannot rule out the potential for bias due to uncontrolled confounding.

Fourth, our analysis relied on a small sample size, and the representativeness of our final sample is unclear. Although phthalate metabolite concentrations did not differ significantly between our final sample and a subsample that included 80% of the initial sample of eligible mothers, women in the final sample were of higher socioeconomic status. The school-age CBCL we employed was normed using a national sample of 1,753 children between the ages of 6 and 18 years (Achenbach and Rescorla 2001). The *T*-score percentiles we observed suggest that our sample had slightly more behavioral problems than the normative sample. The prenatal phthalate metabolite concentrations we observed in this population were all lower than those reported in Wyatt et al. (2012), yet were comparable to those we previously observed in a nationally representative sample of women of reproductive age (Kobrosly et al. 2012).

## Conclusions

Our results suggest associations between exposure to certain phthalates in late pregnancy and behavioral problems, many of which appeared to be specific to boys or stronger in boys than girls. Given the few studies on this topic and the methodological and study population differences between these studies, additional research is clearly warranted.

## Supplemental Material

(190 KB) PDF
