# Supplementary material for: Prenatal Phthalate Exposures and Neurobehavioral Development Scores in Boys and Girls at 6–10 Years of Age
Source: Environ Health Perspect. 2014 Feb 21;122(5):521–8. doi: 10.1289/ehp.1307063 (PMC4014764; doi:10.1289/ehp.1307063)
Supplement: (190 KB) PDF [file ehp.1307063.s001.pdf]

Supplemental Material

Prenatal Phthalate Exposures and Neurobehavioral Development Scores in Boys and Girls at 6-10 Years of Age

Roni W. Kobrosly, Sarah Evans, Amir Miodovnik, Emily S. Barrett, Sally W. Thurston,

Antonia M. Calafat, and Shanna H. Swan

Table of Contents **Page**

Table S1. Partial Pearson correlation matrix of phthalate metabolites in final sample 2

Table S2. Comparison of demographic and phthalate distributions in initial sample of eligible mothers and final sample 3

Table S1. Partial Pearson correlation matrix of phthalate metabolites in final sample [correlation (p-value)].[a]

**Metabolite**	**ln(MEHP)**	**ln(MEHHP)**	**ln(MEOHP)**	**ln(MiBP)**	**ln(MBP)**	**ln(MBzP)**	**ln(MEP)**
ln(MEHP)							
ln(MEHHP)	0.72 (< 0.001)						
ln(MEOHP)	0.76 (< 0.001)	0.98 (< 0.001)					
ln(MiBP)	0.26 (0.001)	0.29 (< 0.001)	0.30 (< 0.001)				
ln(MBP)	0.22 (0.007)	0.41 (< 0.001)	0.39 (< 0.001)	0.51 (< 0.001)			
ln(MBzP)	0.20 (0.01)	0.32 (< 0.001)	0.31 (< 0.001)	0.46 (< 0.001)	0.55 (< 0.001)		
ln(MEP)	0.071 (0.39)	0.22 (0.005)	0.23 (0.004)	0.17 (0.034)	0.38 (< 0.001)	0.31 (< 0.001)	

[a]Correlations adjusting for creatinine.

Table S2. Comparison of demographic and phthalate distributions [mean ± SD or n (%)] in initial sample of eligible mothers and final sample.[a]

Demographics	Mothers eligible for follow-up (n = 359)	Mothers in final sample (n = 153)	p-value for test of difference
Mother's race/ethnicity			
Hispanic/Latina	31 (8.6)	4 (2.6)	
White, non-Hispanic	301 (83.8)	141 (92.2)	
Black	7 (1.9)	5 (3.3)	
Asian	17 (4.7)	3 (2.0)	
Other	3 (0.8)	0 (0)	0.03
Mother's education			
< College	97 (27.0)	23 (15.0)	
≥ College	262 (73.0)	130 (85.0)	0.005
Mother's age at enrollment (years)	30.1 ± 5.1	31.1 ± 4.9	0.04
Family stress during pregnancy	1.7 ± 1.9	1.4 ± 1.7	0.15
**Phthalate levels**			
ln(MEHP)	1.2 ± 1.4	1.3 ± 1.5	0.50
ln(MEHHP)	2.5 ± 1.3	2.6 ± 1.5	0.48
ln(MEOHP)	2.4 ± 1.3	2.4 ± 1.4	0.59
ln(MiBP)	0.9 ± 1.1	0.9 ± 1.1	0.40
ln(MBP)	2.7 ± 1.1	2.6 ± 1.0	0.29
ln(MBzP)	2.0 ± 1.3	1.9 ± 1.3	0.39
ln(MEP)	4.6 ± 1.7	4.3 ± 1.7	0.23

[a]Complete data was not available for the entire sample of eligible mothers (n = 441) so the 359 mothers with complete data are tabled.
